# Mobile Technology Use by People Experiencing Multiple Sclerosis Fatigue: Survey Methodology

**DOI:** 10.2196/mhealth.6192

**Published:** 2017-02-28

**Authors:** Kirsten Van Kessel, Duncan R Babbage, Nicholas Reay, Warren M Miner-Williams, Paula Kersten

**Affiliations:** ^1^ Department of Psychology Faculty of Health and Enviromental Sciences Auckland University of Technology Auckland New Zealand; ^2^ Centre for eHealth Faculty of Health and Environmental Sciences Auckland University of Technology Auckland New Zealand; ^3^ Centre for Person-Centred Research Faculty of Health and Environmental Sciences Auckland University of Technology Auckland New Zealand; ^4^ Department of Rehabilitation School of Health Sciences University of Brighton Brighton United Kingdom

**Keywords:** mobile phone technology, multiple sclerosis, app, fatigue, symptoms, cognitive behavioral therapy, intervention

## Abstract

**Background:**

Fatigue is one of the most commonly reported symptoms of multiple sclerosis (MS). It has a profound impact on all spheres of life, for people with MS and their relatives. It is one of the key precipitants of early retirement. Individual, group, and Internet cognitive behavioral therapy–based approaches to supporting people with MS to manage their fatigue have been shown to be effective.

**Objective:**

The aim of this project was to (1) survey the types of mobile devices and level of Internet access people with MS use or would consider using for a health intervention and (2) characterize the levels of fatigue severity and their impact experienced by the people in our sample to provide an estimate of fatigue severity of people with MS in New Zealand. The ultimate goal of this work was to support the future development of a mobile intervention for the management of fatigue for people with MS.

**Methods:**

Survey methodology using an online questionnaire was used to assess people with MS. A total of 51 people with MS participated. The average age was 48.5 years, and the large majority of the sample (77%) was female.

**Results:**

Participants reported significant levels of fatigue as measured with the summary score of the Neurological Fatigue Index (mean 31.4 [SD 5.3]). Most (84%) respondents scored on average more than 3 on the fatigue severity questions, reflecting significant fatigue. Mobile phone usage was high with 86% of respondents reporting having a mobile phone; apps were used by 75% of respondents. Most participants (92%) accessed the Internet from home.

**Conclusions:**

New Zealand respondents with MS experienced high levels of both fatigue severity and fatigue impact. The majority of participants have a mobile device and access to the Internet. These findings, along with limited access to face-to-face cognitive behavioral therapy–based interventions, create an opportunity to develop a mobile technology platform for delivering a cognitive behavioral therapy–based intervention to decrease the severity and impact of fatigue in people with MS.

## Introduction

New Zealand has a high prevalence of multiple sclerosis (MS). In 2006, the age-standardized prevalence of MS among the general population of New Zealand was 71.9 per 100,000 [1]. In contrast, the age-standardized prevalence of MS in Māori has remained constant at 17.5 per 100,000 [1]. Between 1968 and 2006, the disease frequency for MS in New Zealand has increased by nearly 90%, from 37.8 in 1968 to current level of 71.9 [2]. During the same 38-year period, the gender ratio essentially remained constant.

One of the most commonly reported symptoms of MS is fatigue, affecting more than 80% of patients [3]. Although often identified as a symptom of MS, fatigue is often not treated, perhaps because it typically appears unrelated to the severity of the central disease process [4]. MS fatigue differs from tiredness experienced by healthy people in both severity and impact. MS fatigue has a profound effect on all spheres of life [3,5] for people with MS, their relatives, and the nursing staff [6,7]. Fatigue is one of the key precipitants of early retirement [8,9].

Little is known about levels of fatigue severity and the impact experienced by people with MS fatigue in New Zealand [10]. Self-report measures of fatigue can be an appropriate assessment option given the subjective experience of the symptom. The advantage of self-report measures are that they are generally short, widely available, easy for the patient to understand, and require little training by the assessor [11]. Additionally, self-report measures of fatigue have concurrent validity and are acceptable to people with MS [12]. There is, however, large variation and inconsistency across studies measuring the severity and impact of MS fatigue, which might be partly due to different measures used [13-16]).

At present, the range of measurement options means assessors need to be clear about the aspects of fatigue they intend to measure (eg, severity or impact of fatigue), in which population and for what purpose, in order to select the most relevant self-report measure. There is a paucity of research regarding which measure of fatigue is the most appropriate under differing conditions. A more concise definition of fatigue is needed, along with a high-quality measurement instrument. To attempt to address these issues for people with MS, the Neurological Fatigue Index for MS was developed [16]. This scale was designed to conform to the Rasch measurement model [17] and rigorously tested to determine its reproducibility. The scale can be used with people with MS of “any age, sex, and duration (of MS symptoms)” [18]. The minimum clinically important difference for the Neurological Fatigue Index for MS was found to be small (2.49 of a 30-point range), such that changes in the physical, cognitive, summary, and nocturnal sleep scales were aligned with the respondents’ perceived changes in fatigue, and most importantly, the resultant scores showed no change when none was perceived [18].

The 13-item, self-report measure Fatigue Symptom Inventory was originally designed to measure the intensity and duration of fatigue and its interference with quality of life in breast cancer patients [19]. Although the Fatigue Symptom Inventory has not been validated for MS, Hann et al [19] suggest that the Fatigue Symptom Inventory could be used to evaluate the physical and psychological characteristics of fatigue and quality of life across groups of patients with different diagnoses, and it has been used extensively in research with MS patients.

Along with challenges in measuring severity and impact of MS fatigue and the lack of New Zealand data, there have been challenges in terms of delivering interventions for this group of people. Cognitive behavioral therapy interventions for MS fatigue, delivered in individual, group, or Internet programs, have been found to be effective [20-25]. However, cognitive behavioral therapy interventions delivered face-to-face are not readily available due to the lack of people with this training working in MS-related services and limited health resources [10].

In New Zealand, a recent survey suggests 86% of people use the Internet, of whom 91% have access to broadband [26]. A key limitation of Internet-based cognitive behavioral therapy programs for MS fatigue is that the person needs to be in front of a computer. However, the number of people using smartphones that have access to the Internet is increasing [26], providing a fruitful opportunity for health applications. The key advantages of mobile phone technologies include the ability to provide an individual level of support to change health behaviors and improve disease management, allowing temporal synchronization of the intervention delivery and allowing the intervention to claim people’s attention when it is most relevant or in the best context [27]. There is increasing evidence that text messages and other smartphone technology are effective in drug adherence, improved diabetes self-management [28], and cessation of smoking interventions [29]. However, other than wearable sensors including accelerometers, gyroscopes, and pressure-sensitive textiles [30], there is limited evidence for the use of smartphone technology in people with MS [31-33].

The aim of the current project was to survey people with MS to (1) review the types of mobile devices people with MS use or would consider using for a health intervention, (2) identify the level of Internet access they have, and (3) characterize the levels of fatigue severity and their impact experienced by the people in our sample to provide an estimate of fatigue severity of people with MS in New Zealand. The ultimate goal of this work was to guide the future development of a mobile intervention for the management of fatigue for people with MS.

## Methods

### Questionnaire

A positivist paradigm theoretical framework and survey methodology using a questionnaire was used. The questionnaire included the following:

1. Collection of basic demographic data to enable the contextualization of the data and duplicate checking

2. Assessment of fatigue severity measured using the Neurological Fatigue Index for MS [16]

3. Assessment of fatigue impact measured with the Fatigue Symptom Inventory, a 13-item self-report measure designed to assess the severity, frequency, and daily pattern of fatigue, as well as its perceived interference with quality of life [34]

4. Questions on mobile phone and Internet usage derived from the New Zealand World Internet Project [26] (approval for this use was obtained)

### Recruitment

People with MS were initially contacted via email by the Multiple Sclerosis Society of New Zealand. The study used Dillman’s [35] tailored survey design, which has been shown to result in high response rates. It was not assumed that participants had access to the Internet. A link to a website was provided in the email, where participants could either complete the survey online, request a call from the researcher to carry out the survey by telephone, request a hard copy of the survey that they returned by post, or request an electronic copy of the form by email.

### Statistical Analysis

All analysis was undertaken using SPSS Statistics (2015, IBM Corp) software. The questionnaire data were analyzed as follows:

1. Basic demographic data were checked for duplicate form submissions. We planned to exclude any duplicate questionnaires from the analysis.

2. Fatigue severity was calculated by summing relevant items of the Neurological Fatigue Index for MS summary, physical, diurnal sleep, nocturnal sleep, and cognitive scales. The raw ordinal data were converted to interval-level data using the conversion table set out by Mills and colleagues [16]. Descriptive statistics (means, SDs, ranges) were then calculated.

3. Fatigue impact, as measured by the Fatigue Symptom Inventory, was analyzed descriptively (median, interquartile range, range) for the following subscales: most severe fatigue, least severe fatigue, average fatigue, present fatigue, and fatigue interference with daily life activities.

4. Internet and mobile phone usage was analyzed using frequencies and cross-tabulations by key demographic variables (age, gender, area, and ethnicity).

### Ethics Approval

Ethics approval for the study was obtained from the Auckland University of Technology Ethics Committee (approval number 15/99).

## Results

In total, 51 people with MS took part in the study. We cannot comment on the response rate as recruitment was via social media. The mean age of the participants was 48.5 (SD 12.8) years and ranged from 26 to 71 years. A large majority (39/51, 77%) were female, 16% (8/51) were male, and the majority were New Zealand European. The demographic distribution of the respondents is shown in Table 1. Of the 51 respondents, 38 (75%) lived within an urban environment.

Findings of the levels of fatigue severity and their impact experienced by participants are shown in Table 2. On average, people suffered from significant levels of fatigue as measured with the summary score of the Neurological Fatigue Index (mean 31.4, SD 5.3, range 15-40; maximum possible score is 40). The highest fatigue scores were in the physical subcategory.

The Fatigue Symptom Inventory [24,34] is designed to assess the severity, frequency, and daily pattern of fatigue as well as its perceived interference with quality of life (see Table 3).

**Table 1:** Demographic characteristics of participants (N=51).

Characteristics		
Age (years), mean (SD)		48.5 (12.8)
Range of cohort (years)		26-71
**Gender, n (%)**		
	Female	39 (77)
	Male	8 (16)
	Missing data	4 (8)
**Ethnicity, n (%)**		
	New Zealand European	39 (77)
	European	7 (14)
	Australian	1 (2)
	Missing data	4 (4)
**Employment, n (%)**		
	Full-time	14 (28)
	Part-time	7 (14)
	Not employed	24 (47)
	Missing data	6 (12)

**Table 2:** Descriptive statistics of the Neurological Fatigue Index (n=47).

Subscale	Mean	SD	Minimum	Maximum
Physical	25.8	4.7	10.0	32.0
Cognitive	11.1	2.8	4.0	16.0
Relief by diurnal sleep or rest	16.7	2.7	11.0	23.0
Abnormal sleep and sleepiness	14.6	2.8	9.0	20.0
Summary	31.4	5.3	15.0	40.0

**Table 3:** Descriptive statistics of the Fatigue Symptom Inventory (n=47).

		Mean (SD)
**Fatigue severity in past week**		
	Maximum fatigue in past week	7.06 (1.87)
	Minimum fatigue in past week	3.28 (1.87)
	Average fatigue in past week	5.09 (1.90)
	Fatigue right now	5.19 (2.48)
	Subtotal	5.15 (1.74)
**Fatigue interference with activities in past week**		
	General activity	5.28 (2.65)
	Ability to bathe and dress yourself	2.38 (3.00)
	Normal work activity	4.72 (2.95)
	Ability to concentrate	4.43 (2.88)
	Relations with other people	3.81 (2.54)
	Enjoyment of life	5.26 (2.62)
	Mood	4.72 (3.01)
	Subtotal	4.37 (2.29)
**Frequency of fatigue in the past week**		
	Number of days fatigued in past week	5.72 (1.81)
	How much of the day were you fatigued	5.45 (2.48)
	Subtotal	5.59 (2.14)

Lower scores denote less acute problems with fatigue. The total mean score of fatigue severity in the past week was 5.59 (SD 1.9). In total, 84% (43/51) of respondents scored on average more than 3 on the fatigue severity questions, implying significant fatigue. The total mean score for fatigue interference with activities during the past week was 4.37 (SD 2.3). Fatigue frequency during the past week was 5.45 (SD 2.5). The majority of participants reported their fatigue to be worst in the afternoon (22/51, 43%), but a sizeable group said there was no consistent pattern to their fatigue (14/51, 28%). The majority of participants (37/51, 73%) reported the use of strategies to alleviate fatigue (eg, timing of certain activities, managing stress, pacing and resting).

Mobile phone usage was high, with 86% (44/51) of respondents reporting that they have a mobile phone; 41% (44/51) use an iPhone, 39% (20/51) use an Android phone, 2% (1/51) use a Windows phone, and 4% (2/51) use a Blackberry. In addition, 9% (5/51) have access to 2 phones.

Mobile phone apps were used by 75% (38/51) of respondents. A total of 6% (3/51) stated they didn’t know, and 20% (10/51) did not answer the question. Along with mobile phones, people with MS reported using a range of other mobile devices, including laptops (32/51, 63%) and tablets like iPad (25/51, 49%), iPod Touch (6/51, 12%), iPad Mini 4 of 51 (4/51, 8%), and e-book readers (14/51, 28%). Half of respondents (25/51, 49%) had access to 2 mobile devices. Finally, 1 participant could not afford a tablet and 1 person reported having no need for one.

When asked where they were able to access the Internet, 92% (47/51) reported accessing the Internet from home. Other locations where participants accessed the Internet included work, locations outside the home, and other homes (Table 4). Daily Internet usage was commonly reported by participants with an average use of 2 hours, 23 minutes per day. One-quarter (13/51, 26%) of participants did not access the Internet at all.

**Table 4:** Locations where participants have Internet access.

Location	Percentage
Home	92
Work	33
School	2
Internet café	4
Other homes	16
Library	6

Some participants reported that mobile phone or app use was negatively affected by their symptoms, such as visual problems (6/51, 12%) and weakness (15/51, 29%). Examples of visual problems included trouble focusing, optic neuritis, and blind spots. People also reported other symptoms of MS that could affect their use of such technology, such as eyestrain, general fatigue, numbness in the fingers, numbness of 1 side of the body, excessive tremor in hands and fingers, and 1 person reporting difficulty with voice recognition training problematic because of slurred speech.

Only a small percentage of participants (4/51, 8%) reported the use of special devices to access mobile technology. Such special devices included onscreen virtual keyboards (12/51, 23%), alternative mouse systems (3/51, 6%), voice recognition (3/51, 6%), and a screen magnifier (2/51, 4%).

In summary, New Zealand survey participants with MS reported high levels of both fatigue severity and fatigue impact. Responses also indicated that the large majority of participants have a mobile device, use apps, and have access to the Internet.

## Discussion

### Principal Findings

This study assessed levels of fatigue severity and their impact experienced by people with MS in New Zealand. Fatigue severity was measured using the Neurological Fatigue Index for MS. Fatigue significantly affected nearly all of those who took part in the study; both physical and cognitive fatigue affected their quality of life. Of the subscale categories surveyed, fatigue predominantly affected motor function and sleep patterns, findings which are in line with the studies of Thomas et al [23] and Carnicka et al [36]. Difficulties with motor function and sleep often lead to anxiety and depression [5,23,36,37]. Disruption of melatonin circadian rhythm production and lower waking cortisol levels have been linked with higher disability and fatigue scores in MS patients [38,39]. The majority of participants in this study experienced more fatigue in the afternoon. This is consistent with subjective reports of increasing cognitive fatigue during the day by MS patients [40].

On the Fatigue Symptom Inventory, the majority (43/51, 84%) of respondents reported this to be severe. Such levels of fatigue interfered significantly with people’s day-to-day activities, results that are consistent with those of Mills and Young [41], who also found relationships between fatigue and disability, disease type, and sleep.

Given that both fatigue severity and fatigue impact were reported to be severe and significant, there is an important need for accessible evidence-based interventions for people with MS. The possibility of using mobile technology to deliver such an intervention could be a solution to the current health environment of scarce resources because people with MS appear to be open to smartphone use in health care and have reported many potential benefits [33,42]. This study obtained some useful findings in regard to access and use of mobile technologies by people with MS fatigue. The majority of participants in this survey were open to smartphone use, and only a small number of participants (2/51, 4%) reported MS symptoms that restricted their use of mobile phone apps. Furthermore, most repondents in the study had a mobile phone and access to the Internet at home, suggesting that a sufficient platform exists to develop a mobile app to deliver a cognitive behavioral therapy–based intervention for MS fatigue.

The use of mobile technology in providing an intervention for MS fatigue would need to consider MS disabilities which may limit dexterity, and the design and implementation of eHealth apps should be tailored to the patients’ individual needs [43].

There are a number of general benefits associated with mobile technologies [44], which are also relevant to the population of interest. The application of inexpensive wireless technologies such as accelerometers and gyroscopes combined with Internet-based or smartphone apps offers researchers and clinicians a viable method of monitoring patients with MS. Such feedback and biofeedback could improve self-management and home-based rehabilitation [30,45]. Mobile technologies can lower the costs and burden of travel to clinic-based assessments, remove the subjectivity of self-reporting, and improve the capture of data with greater accuracy and precision regarding the daily impairment, disability, and functioning of patients with MS [30,45]. Mobile technology also permits accessing interventions at times of the day when the user is least affected by fatigue [40].

### Limitations and Future Directions

With such a small survey (51 respondents) and unknown response rate it is difficult to ascertain conclusively the nature and severity of symptoms of MS fatigue or the use of mobile devices. However, the sample is reflective of the typical epidemiology of people with MS (eg, predominantly female and white). The findings of the study will be used to investigate the benefits of a mobile technology app to deliver a cognitive behavioral therapy–based intervention for the management of MS fatigue.

### Conclusion

This survey has demonstrated that New Zealand respondents with MS experienced high levels of both fatigue severity and fatigue impact. The majority of participants have a mobile device and access to the Internet. These factors, along with limited access to face-to-face cognitive behavioral therapy–based interventions, create an opportunity to develop a mobile technology platform for delivering a cognitive behavioral therapy–based intervention to improve the severity and impact of fatigue in people with MS.

## Abbreviations

MS: multiple sclerosis
